# The effectiveness of a stratified care model for non-specific low back pain in Danish primary care compared to current practice: study protocol of a randomised controlled trial

**DOI:** 10.1186/s13063-018-2685-5

**Published:** 2018-06-08

**Authors:** Lars Morso, Berit Schiøttz-Christensen, Jens Søndergaard, Nils-Bo de Vos Andersen, Flemming Pedersen, Kim Rose Olsen, Morten Sall Jensen, Jonathan Hill, David Høyrup Christiansen

**Affiliations:** 1 Centre for Quality, Region of Southern Denmark, P.V. Tuxensvej 5, 5500 Middelfart, Denmark; 2 Department of Regional Health Research, University of Southern Denmark, Odense, Denmark; 3 Spine Centre of Southern Denmark, Odense, Denmark; 4 Research Unit of General Practice, University of Southern Denmark, Odense, Denmark; 5 Department for Health Provision, Region of Central Denmark, Viborg, Denmark; 6 Physiothery Primary Care Consultant, Region of Southern Denmark, Vejle, Denmark; 7 Department of Business and Economics, COHERE, University of Southern Denmark, Odense, Denmark; 8 Institute for Primary Care and Health Sciences, Keele University, Staffordshire, UK; 9 Department of Occupational Medicine, Regional Hospital West Jutland, University Research Clinic, Herning, Denmark

**Keywords:** Stratified care, STarT back tool, Randomised controlled trial, Cost effectiveness

## Abstract

**Background:**

Prior studies indicate that stratified care for low back pain results in better clinical outcome and reduced costs in healthcare compared to current practice. Stratified care may be associated with clinical benefits for patients with low back pain at a lower cost, but evidence is sparse. Hence this study aims to evaluate the clinical effects and cost-effectiveness of stratified care in patients with non-specific low back pain compared to current practice.

**Methods/design:**

The study is a two-armed randomised controlled trial in primary care in the Regions of Southern and Central Denmark (2.5 million citizens). Patients with non-specific low back will be recruited by paticpating GPs. Patients are randomised to either (1) stratified care or (2) current practice at participating physiotherapy clinics. In the stratified care arm, the intervention is based on the patient’s STarT Back Tool classification and trained accordingly, whereas physiotherapists in the current pratice arm are blinded to the STarT score. Primary outcomes in the trial will be group differences in time off work, improvement in LBP disability measured by the Roland Morris Disability Questionnaire (RMDQ) and patient-reported global change. Secondary measures will be pain intensity, patient satisfaction, data on patient healthcare resource utilisation and quality-adjusted life year based on the EQ-5D-5L.

**Discussion:**

Stratified care that effectively targets treatment to relevant sub-groups of patients has potentially great impact on the treatment pathways of low back pain. Thus, if effective, this could result in better patient outcomes and at the same time reduce the costs for treatment of low back pain.

**Trial registration:**

ClinicalTrials.gov, NCT02612467. Registered on 16 November 2015.

**Electronic supplementary material:**

The online version of this article (10.1186/s13063-018-2685-5) contains supplementary material, which is available to authorized users.

## Background

Emerging evidence indicates that stratified care of low back pain (LBP) may result in better clinical outcome and reduced healthcare costs, compared to usual care [1]. Stratified care is a way to manage the complexity of non-specific LBP (NSLBP) [2]. This approach involves a simple nine-item screening questionnaire, the STarT Back Tool (SBT). The SBT has been translated and cross-culturally validated in a Danish-speaking population [3, 4]. Using this tool, patients are assigned to one of three subgroups, based on modifiable indicators of prolonged LBP, and then manage according to a matched treatment pathway [5].

Patients are classified into low-risk, medium-risk and high-risk subgroups based on the SBT score. Conceptually low-risk patients are likely to have a good outcome with a one-off good quality consultation consisting of reassurance, analgesia and high-quality health information about good self-management. Patients at medium risk are likely to benefit from evidence-based physiotherapy treatment focusing on reducing pain and disability and enabling patients to manage ongoing or future episodes of LBP. High-risk patients are to receive a more comprehensive combined physical and psychosocial intervention, according to this stratified care model termed as psychologically informed physiotherapy (PIP) [5].

Studies from the UK have shown stratified care to be superior to usual care in primary care LBP patients. Furthermore, the stratified care model resulted in a 50% reduction in LBP-related sickness absence and lower healthcare costs compared to current best practice [6]. Thus, stratified care may be associated with clinical benefits for LBP patients in primary healthcare at lower costs. However, still evidence is sparse and healthcare and social systems may vary between countries (e.g. in the way reimbursement and referral systems works). Therefore, replication of the UK findings is warranted before implementation in Danish healthcare settings can be recommended. The aim of this study is to evaluate the clinical effects and cost-effectiveness of a stratified care model in patients with NSLBP compared to current practice in a Danish primary healthcare setting.

## Methods, design and setting

**Fig. 1:**
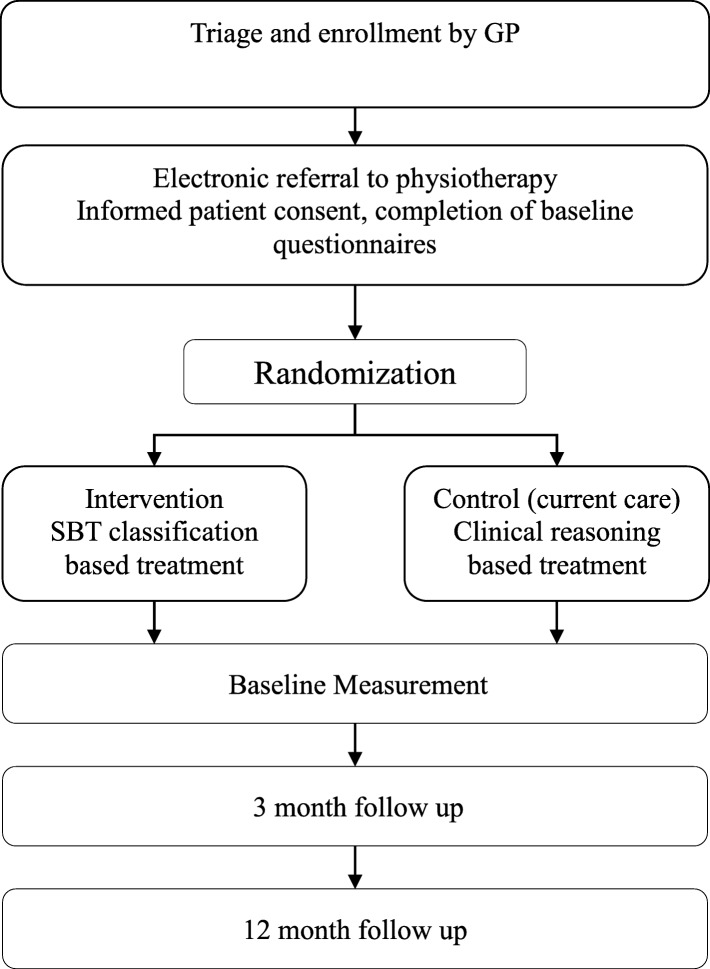
Flow chart of inclusion, treatment and follow-up

**Fig. 2:**
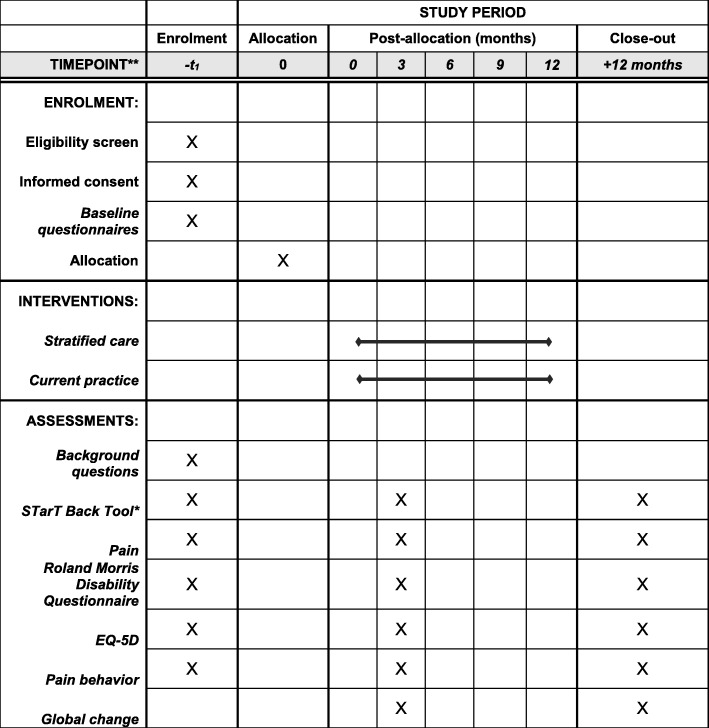
SPIRIT. Schedule of enrolment, assessment and interventions

We will perform the study as a two-armed randomised controlled trial (RCT) in primary healthcare from December 2015 to December 2017 in the Regions of Southern and Central Denmark. Follow-up points will be at three and 12 months (for further information, see Fig. 1). All data collection and randomisation procedures will be administered through an existing web-based research portal (https://trialpartner.clin.au.dk). Patients are recruited by general practitioners (GP) in ten municipalities across Southern and Central Denmark Regions; a total of 42 GPs practices and 21 physiotherapy clinics participate. At the initial or second consultation (see Fig. 2), the GP will assess, triage and electronically refer patients to physiotherapy according to their normal practice specified in the recommendations of the Danish Society of General Practice [7]. Patients who decline referral will not be eligible for the study and will follow the usual clinical trajectory.

## Inclusion and exclusion criteria

The inclusion criteria are: are patients diagnosed with NSLBP and found relevant for referral to physiotherapy by the GP; age 18 years and above; and understand Danish language. Patients with or without contributing leg pain can be included in the study.

The exclusion criteria are: serious pathology (malignancy, inflammatory arthritis, etc.); serious nerve root compression (cauda equine, paresis < 3), influential co-morbidity; psychiatric illness; personality disorder; spinal surgery during the last six months; pregnancy; or already receiving physiotherapy treatment for the present episode.

## Recruitment

### Clinicians

The recruitment of GPs and clinicians will be from the same local area and cover different geographical parts of the Regions of Southern and Central Denmark. Local information meetings will be held at GP practices and the physiotherapists will be trained/instructed to deliver either stratified care (intervention) or current practice (control).

### Patients

Participating patients are recruited when consulting their GP for an episode of LBP. The GP assesses and diagnoses/triages the patient according to their clinical guideline. Patients diagnosed with NSLBP and who are eligible for the project according to the inclusion and exclusion criteria are asked for their interest in participation. If interested, the patients will be handed written information and asked for their phone number for later contact. Finally, they are electronically referred to physiotherapy (via the usual electronic referral site ‘RefHost’). The project secretary is continuously informed of new potential participants by the Trial partner system.

Within 1–2 days, interested patients are contacted by the project secretary by telephone. The project secretary repeats information on study details, checks inclusion/exclusion criteria and answers any questions regarding the written information. If the patient is still interested in participating, an email containing a hyperlink is sent on an encrypted line. When activating the link, a consent form appears to be signed with a digital signature.^1^ This form is automatically returned and the patient can now complete the baseline questionnaires.

## Randomisation

After receiving the patient’s questionnaire and signed consent, the project secretary randomises the patient into one of the two treatment arms stratified for city and SBT sub-group using computer-generated random number sequence (Fig. 2). By randomisation, the patients in the study are ensured to receive treatment based at least on best practice. After randomisation, the project secretary contacts the relevant physiotherapy clinic to ensure initiation of treatment. At three-month and 12-month follow-up, questionnaires are electronically sent to the patient. Electronic reminders are sent after 14 days, followed by a phone reminder seven days later. Randomisation, data collection and reminders are administrated by an online clinical database developed for the study (Additional file 1).

## Intervention

### Stratified care group (intervention group)

A structured standardised physical assessment will be performed. This includes patient history concerns and treatment expectations and a core set of relevant physical tests (e.g. neurological examination if relevant, back pain movements and testing for a directional preference). The results of the clinical assessment, scores from questionnaires and the SBT sub-group classification in combination are used as clinical guidance and the appropriate matched treatment will be delivered accordingly. The treatment is delivered by physiotherapists who have received adequate training to deliver the stratified care. The training will consist of a five-day course. The training course includes approaches on how to address patient beliefs, attitudes and behaviour. In interactive workshops, training sessions and role plays, these approaches are tested and practised. The deliverance of training will be undertaken by a highly skilled physiotherapist who was one of the developers behind the development and education in the English STarT Back Trial [8]. To ensure maintenance of skills and adherence to the protocol, clinical training and support programmes for the physiotherapists in the study intervention group will mirror the STarT Back Trial in the UK [4]. All patients receive reassuring information. The topics of reassurance are guided by the SBT score and the clinical assessment. Patients are encouraged to maintain or return to normal activity if possible. An informative back book and an online film are provided to supplement messages from the consultation. Advice and information about medication, further examinations, work, prognosis, future episodes, pain-coping strategies and individual patient concerns will be addressed. The main components of the stratified care are provided below:

- Low-risk intervention: patients in the low-risk group will receive a good-quality, evidence-based consultation. Minimal intervention with emphasis on relevant information and activity will be relevant for this group. Onwards referral, investigation or further treatment is not recommended unless the physiotherapist finds it highly relevant.
- Medium-risk intervention: in addition to the above, patients will receive an evidence-based standardised package of individualised treatment focusing on restoring function (targeting disability, back, leg and co-morbid pain). The medium-risk group will also receive an intervention that focuses on the prevention of future back pain episodes.
- High-risk intervention: in addition to the intervention described above, these patients will receive individualised PIP aiming to reduce pain and disability and improve psychological functioning (where possible). Treatment is centred around value-based goal-setting and is facilitated by the use of cognitive and/or behavioural techniques that focus on improvement in quality of life and enables patients to manage ongoing and/or future episodes of LBP [8].

### Current practice (control group)

In contrast to the stratified care group, the decisions on whether patients should receive further physiotherapy treatment will be solely based on clinical judgement, clinical need and patient preferences. The physiotherapist has no access to any of the baseline patient questionnaires. Current best practice will be delivered by well-educated and qualified physiotherapists. We register the educational level of the physiotherapists to secure uniformity between the groups. A standardised physical assessment will be conducted. The main treatment modalities to be used should broadly reflect current practice in Danish primary care (e.g. Manual Therapy, Mechanical Diagnosis and Therapy, exercise, acupuncture, usual advice and reassurance).

## Outcome measures

A core set of standardised and internationally recommended outcome measures will be applied in the trial [9]. The primary outcomes in the trial will be improvement in LBP disability measured by the Roland Morris Disability Questionnaire (RMDQ) [10], group differences in time off work and patient-reported global change. Time off work is considered a complicated measure [2], but standardised patient-reported data from the project database allows us to monitor short-term sick leave (measured in days) and information from the Danish National Register on Public Transfer Payments (DREAM) makes it possible to monitor long-term sick leave (> 2 weeks of consecutive absence) and other related social benefits such as pensions [11]. Secondary outcomes will be pain intensity, patient satisfaction, data on patient healthcare resource utilisation and EQ-5D-5 L [12, 13].

It is furthermore planned to monitor the stratified model’s effectiveness on appropriateness of referrals and the ability to reduce referrals to secondary care. The outcome measures here will be numbers of referrals to secondary care using the stratified model compared to current practice, increased detail and usefulness of referrals sent to secondary care, and numbers of consultations in secondary care for patients initially exposed to stratified care compared to current practice. Data for these analyses will come from the two central secondary care spine centres in the regions.

## Analyses

Analysis will be conducted using intention-to-treat. Scores of primary and secondary continuous outcomes are compared between groups using appropriate descriptive statistics, mixed multi-level linear models and Generalised Estimating Equations models for categorical outcomes at three and 12 months taking into account cluster effects of the treating physiotherapist [14]. Secondary analysis using multiple imputation on missing data and loss to follow-up will be conducted [15, 16]. The health economic analysis will use a decision analytic framework (e.g. Markov-model or/and Decision Tree analysis) to test the cost-effectiveness of stratified care against current practice. We use a healthcare perspective for the cost-effectiveness analysis. Cost data comprise trial patient healthcare-resource use and unit cost from national provider agreements and diagnostic-related groups. Quality-adjusted life years (QALY) are calculated based on the EQ-5D with Danish QALY weights. Relevant sensitivity analyses will be performed to address the robustness of the results. A Budget Impact analysis will be made to describe the budget cost or potential budget savings for the different healthcare payers of a fully implemented SBT in the Regions compared to current practice.

The results of the study will be published according to the Consolidated Standards of Reporting Trials (CONSORT) statement for RCTs international peer-reviewed journals.

## Sample size

In the UK, the STarT Back Trial protocol [17] sample size was calculated on basis of the RMDQ as the primary outcome measure. A difference of 2.5 in change scores on the RMDQ was considered to be a minimum clinically important difference. In this study, we wanted to mirror the UK study; therefore, we used the same RMDQ change score for the sample size calculation. We inserted numbers (mean = 13.2, standard deviation = 5.4) from a prior Danish study [3] into S-13 to calculate a sample size with a power of 80% and significance level at 5%. The calculation showed that 75 patients are needed in each treatment arm to detect an overall 2.5 difference in change scores on the RMDQ between the intervention and current care group. In the study, we also want to be able to analyse the effect in the high-risk subgroup. The prevalence in Danish primary care has earlier been shown to be approximately 25%. With an expected 10% drop out rate, a total of 660 patients will be included in the study.

## Adverse effects

All adverse effects will be registered and reported.

## Discussion

LBP constitutes a global health problem and stratified care that effectively targets treatment to relevant sub-groups of patients has the potential to have great impact on the treatment pathways of LBP. If effective, the results of this study will qualify whether stratified care models may serve as a part of the national Danish implementation strategy for evidence-based LBP care in primary healthcare. Potentially, this could significantly result in better patient outcomes and at the same time reduce the costs for treatment of LBP.

## Trial status

Recruitment is ongoing.

## Additional file

Additional file 1: SPIRIT – checklist. Recommended items to address in a clinical trial protocol. (DOC 118 kb)

## Abbreviations

CONSORT: Consolidated Standards of Reporting Trials
DREAM: Danish National Register on Public Transfer Payments
LBP: Low back pain
NSLBP: Non-specific low back pain
PIP: Psychologically informed physiotherapy
RMDQ: Roland Morris Disability Questionnaire
SBT: STarT back tool
SPIRIT: Standard Protocol Items: Recommendations for Interventional Trials
STarT: Stratification targeted treatment

## Footnotes

1
https://www.nemid.nu/dk-da/
